# Giving syphilis and gonorrhea to friends: Using in-person friendship networks to find additional cases of gonorrhea and syphilis

**DOI:** 10.21203/rs.3.rs-22332/v2

**Published:** 2020-07-31

**Authors:** Janet E. Rosenbaum, Jacky Jennings, Jonathan Ellen, Laurel Borkovic, Jo-Ann Scott, Charleen Wylie, Anne Rompalo

**Affiliations:** SUNY Downstate Health Sciences University; Johns Hopkins University School of Medicine; Laborador Health; Johns Hopkins University School of Medicine; Johns Hopkins University School of Medicine; Johns Hopkins University School of Medicine; Johns Hopkins Medicine

**Keywords:** gonorrhea, syphilis, partner notification, contact tracing, bisexuality, sexual orientation, labeling, self concept

## Abstract

**Background:**

Syphilis and gonorrhea reached an all-time high in 2018. The resurgence of syphilis and gonorrhea requires innovative methods of sexual contact tracing that encourage disclosure of same-sex sexual contacts that might otherwise be suppressed. Over 75% of Grindr mobile phone application users report seeking “friendship,” so this study asked people diagnosed with syphilis and gonorrhea to identify their friends.

**Methods:**

Patients at the two Baltimore sexually transmitted infection (STI) clinics and the Baltimore City Health Department were asked 12 questions to elicit members of their friendship networks before eliciting sexual networks. The study included 353 index cases and 172 friendship contacts, yielding a friendship network of 331 non-isolates (n=331) and sexual-only network of 140 non-isolates. The data were plotted and analyzed using exponential family random graph analysis.

**Results:**

Eliciting respondents’ in-person social contacts yielded 12 syphilis cases and 6 gonorrhea cases in addition to the 16 syphilis cases and 4 gonorrhea cases that would have been found with sexual contacts alone. Syphilis is clustered within sexual (odds ratio=2.2, 95% confidence interval (1.36, 3.66)) and social contacts (OR=1.31, 95% CI (1.02, 1.68)). Gonorrhea is clustered within reported social (OR=1.56, 95% CI (1.22, 2.00)) but not sexual contacts (OR=0.98, 95% CI (0.62, 1.53)).

**Conclusions:**

Eliciting friendship networks of people diagnosed with syphilis and gonorrhea may find members of their sexual networks, drug use networks, or people of similar STI risk. Friendship networks include more diagnosed cases of syphilis and gonorrhea than sexual networks alone, especially among populations with many non-disclosing men who have sex with men (MSM) and women who have sex with women (WSW). Future research should evaluate whether this friendship network method of contact tracing can be implemented by adapting automated mobile phone COVID-19 contact tracing protocols, if these COVID-19 contact tracing methods are able to maintain anonymity and public trust.

## 1. Background

The 2018 resurgence of syphilis and gonorrhea to the highest levels in over two decades concomitant with resource constraints requires innovative methods of control (1). Traditionally, disease intervention specialists from state and local health departments review sexually transmitted infection (STI) cases to conduct sexual contact tracing, an approach known to yield incomplete information because index cases may not remember or may conceal some sexual partners (2). Contemporary partner notification approaches have used messaging through the internet and/or cellular phones, but a systematic review found low utilization and unclear impact on the number of identified cases (3,4): internet and text messaging partner notification for HIV exposure found eight additional HIV cases for 259 index cases (5). Failure to identify and trace contacts leaves likely syphilis and gonorrhea cases unidentified, untreated, and available to contribute to the further spread of the disease. Past studies of STI clinic patients have found that they often choose sexual partners from within their social, personal, and drug networks, and half of partners in urban Baltimore live within one mile (6). Exploring patients’ broader social contexts may be an effective method of discovering additional cases of syphilis and gonorrhea through identifying unnamed sexual partners or people within the same sexual network.

Past research has used digital social media such as Facebook to contact sexual partners named in a traditional contact tracing process (7) and has augmented reported sexual contacts with reports of social contacts from in-person interviews (2) and venue attendance (8). Partner notification yields incomplete information, especially for men who have sex with men (MSM), and remains challenging (9, 10). In 2006, the US Centers for Disease Control and Prevention (CDC) called for “re-evaluating partner notification (PN) methods for syphilis to determine the relative effectiveness of PN strategies and to ensure that enhanced methods are implemented.” (11). However, the current partner notification guidelines suggest asking only for sexual contacts (10).

In this study we test the power of social and personal network analysis in explaining syphilis transmission. These findings can be applied to evaluate a new method of syphilis control by comparing the effectiveness of detecting early infectious syphilis cases by screening the friendship network members of syphilis and gonorrhea index cases compared to standard sexual partner notification techniques. The index cases are the patients diagnosed with syphilis and gonorrhea in the STI clinic and asked to provide their social and sexual contacts. This study elicited information that may otherwise be hidden by asking respondents to reveal their social contacts: the most important people in their lives, with whom they eat, drink, live, and confide in. Eliciting social contacts may reveal additional syphilis and gonorrhea cases either because the social contacts are from similarly vulnerable populations with similar behavior, sexual networks, and STI risk, or because contacts are actually sexual contacts that the respondent chooses not to reveal.

## 2. Methods

### 2.1 Data

Participants for this study were recruited from the two Baltimore City Health Department STI clinics. In the usual contact-tracing procedure, patients diagnosed with syphilis and gonorrhea were asked at their STI clinic visit to name sexual contacts from their likely window of infectivity for partner notification purposes. For patients infected with gonorrhea or primary syphilis, this window was defined as 3 months prior to the onset of symptoms; for patients infected with secondary syphilis or early latent syphilis, this window was defined as 6 months prior to the onset of symptoms. For this study, patients who were diagnosed with syphilis and gonorrhea at one of the STI clinics were interviewed privately and asked to name members of their friendship networks and given a self-administered questionnaire. The friendship network questions did not concentrate on friends during the likely window of infectivity (Table 1). In order to assure respondents of confidentiality, these interviews were conducted by trained interviewers at a research center located in a brownstone 5 blocks away from one of the two STI clinics.

Between March 2001 to December 2005, 353 index cases diagnosed with gonorrhea or primary, secondary, or early latent (early non-primary non-secondary) syphilis who presented to public health clinics were recruited as part of a social and sexual network study in an urban setting with a high prevalence of STIs. By mistake, seven index cases had not tested positive for either gonorrhea or syphilis; these individuals may have been designated as index cases due to either miscoding of STI results or index case status.

Participants gave genital biologic specimens for syphilis, HIV, gonorrhea and chlamydia testing. Gonorrhea was diagnosed by symptoms, Gram stain, and culture from genitals. Syphilis was diagnosed according to stage. Primary syphilis was diagnosed by presence of genital lesion and evaluation of secretions collected from genital lesion for spirochetes consistent with Treponema Pallidum. Secondary syphilis was diagnosed by symptoms (e.g., fever, chills, myalgias, arthralgias, patchy alopecia, mucous patches) and/or rash, together with reactive serologic tests for syphilis (positive rapid protein reagin (RPR) test) with confirmatory Fluorescent Treponemal Antigen-Absorption test (FTA-ABS). Early latent syphilis was diagnosed by a newly reactive RPR and confirmatory FRA-ABS in the past year; patients with previous reactive RPR/FTA-ABS in the past year, early latent syphilis was defined by documenting a fourfold rise in RPR titer within the past year.

The 353 index cases referred 172 contacts to the study, for a total of 525 participants. The sample size was chosen using a t-test-based power calculation to detect a difference in the population mean of 0.3 infected persons between the friendship networks and sexual networks, with over 90% power. Trained interviewers administered a survey to participants soliciting information on demographics, STI/HIV risk behaviors, and social and sexual contacts. Social contacts were elicited by asking participants 12 questions derived from the Social Support Questionnaire (12) (Table 1), including who would give them a ride to the doctor, who would lend them $25 or more or something valuable, who would visit them if they were in the hospital, former housemates/roommates, whom they eat meals with, whom they drink alcohol with, and whom they discuss important issues with. Respondents were then asked which of the social contacts they have had sex with in the past 6 months. All sexual and social contacts who were named in response to any of the 12 friendship network questions or the 1 sexual network question were asked to participate in the study. The contacts of index cases and contacts of contacts of index cases (sometimes called the first and second tiers) were also asked to refer their social and sexual contacts. Respondents were compensated $10 for the specimen, $35 for the interview, and $10 per enrolled contact.

Each study participant is represented only once in the analysis, irrespective of STI status and connections to other participants. Contacts of different index members with the same first and last name and address were linked and treated as the same individual, allowing graph components to be linked across index members.

For both sexual and social connections, contacts were interviewed using the same questionnaire as the index cases and were tested for syphilis and gonorrhea. Blood and lesion specimens were used for biological strain analysis of Treponema pallidum based on restriction fragment length polymorphism (RFLP) analysis to confirm connections between reported contacts.

### 2.2 Measures

This analysis uses social network analysis, which borrows terminology from graph theory, a branch of discrete mathematics. In graph theory, a graph is a collection of vertices (singular: vertex) and edges between vertices. Vertices are represented as points/dots. Edges may be directed or undirected: a directed edge is represented as an arrow, and a directed edge is represented as a line. A social network represents people as vertices and connections between people as edges in a graph. In social network analysis, the degree of a vertex is the number of edges connected to it. An isolate is a vertex of degree zero: that is, a person without any connections. A non-isolate is a vertex of degree 1 or higher: that is, a vertex with at least one connection.

In this study, vertices were coded according to their attributes including whether the individual was an index case vs. contact, their gender, race, age, and test results for HIV, gonorrhea, chlamydia, syphilis, and syphilis stage. Edges were coded by whether they were sexual or social contacts. The edges were directed, with the direction of the edges indicating nomination. In this case, non-isolates are study participants who had at least one contact come to the STI clinic.

Gender was coded by the interviewer with the item “Record sex as observed.” Interview-perceived gender was the only gender variable collected in the survey, so throughout this paper, “male” means interview-perceived male and “female” means interview-perceived female, so the categories are not equivalent to sex assigned at birth.

Number of sexual partners in the past 90 days was elicited from the survey with the question, “During the past 90 days, with how many different people did you have sex?”

Given racial disparities in STI, with the greatest burden among Black Americans (1), race/ethnicity was coded by the question “Record racial or ethnic background.” Black status was coded as 1 for participants responding “African-American, Black non-Hispanic” or “Black Hispanic” and 0 for participants categorized as “White, non-Hispanic,” “White, Hispanic,” “Asian/Asian-American”, and “Other.”

The number of new cases of syphilis and gonorrhea detected by referral was counted in two ways: the number of total respondents referred by index cases and the number of contacts referred by index cases.

### 2.3 Analysis

The data were cleaned in Microsoft Excel and analyzed using the R igraph package (13). Participants without any connections to other people in the graph (i.e., isolates, vertices of zero degree) were discarded. Some index members had none of their nominated contacts participate but were not degree zero because they were named by another index member. Index members were also named as contacts by other index members: 92 index members were named as social contacts and 72 index members were named as sexual contacts.

After eliminating the 199 isolates (e.g., individuals with no nominated contacts who participated), the associations between the graph structure and respondents’ STI status were found using exponential random graph (p*) models with a tie/no-tie Metropolis-Hastings (14), using the statnet package (15), which is a form of logistic regression. Due to the small number of cases, there were insufficient degrees of freedom to use covariates in the regression analysis.

Differences between groups by STI status were estimated using a permutation test. Continuous variables were plotted using kernel density plots in R stratified by STI status, which offer a high information to ink ratio.

Missing data may bias study results if study participants’ characteristics are associated with data missingness (i.e., whether data are missing). To test whether missingness may cause bias, we tested whether study participants’ characteristics were associated with their contacts’ likelihood of coming to the STI clinic to complete the survey. The degree of each network member is the number of their contacts that came to the STI clinic to complete the survey. To evaluate whether data may be differentially missing according to STI status, we used Fisher’s exact test to test for associations between the degree of each network member and six variables strongly associated with STI status (1): gender (p=0.2), Black or African-American identity (p=0.5), HIV status (p=0.5), chlamydia status (p=0.9), gonorrhea status (p=0.3), and syphilis status (p=0.3). Due to the immune response to STIs, STIs increase the risk of transmitting HIV or contracting HIV, even after treatment. All tests were insignificant at p=0.05 level, implying that data missingness is not related to gender, race, or HIV/STI test results.

To evaluate whether there was a difference in STI test result missingness between index cases and contacts, we tested for missingness with a chi-square test. Missing data for STI tests may result from declining the test or inconclusive STI results. For example, samples of secretions from syphilis lesions cannot be used to test for chlamydia, so a chlamydia test would require an additional sampling procedure, which the participant could refuse; participants may also refuse if they had recently been tested for chlamydia or HIV. Index cases were less likely to be missing HIV test results than contacts (13% vs. 65%, p<0.001), but index cases were more likely to be missing chlamydia results (21% vs. 0%, p<0.001) than contacts were. Index cases and contacts did not differ in the missingness of syphilis and gonorrhea results.

## 3. Results

A total of 353 index cases answered the survey, of whom 180 had syphilis, 153 had gonorrhea, 13 had both syphilis and gonorrhea, and seven had neither. Among the 172 contacts of the index cases, six tested positive for syphilis, four tested positive for gonorrhea, seven tested positive for chlamydia, and four tested positive for previously-diagnosed HIV. The index cases also referred 164 other index cases, of whom six tested positive for syphilis and two tested positive for gonorrhea at their repeat visit to the clinic. The RFLP analysis of syphilis strains confirmed that the social contacts had the same strain.

**Figure 1:**
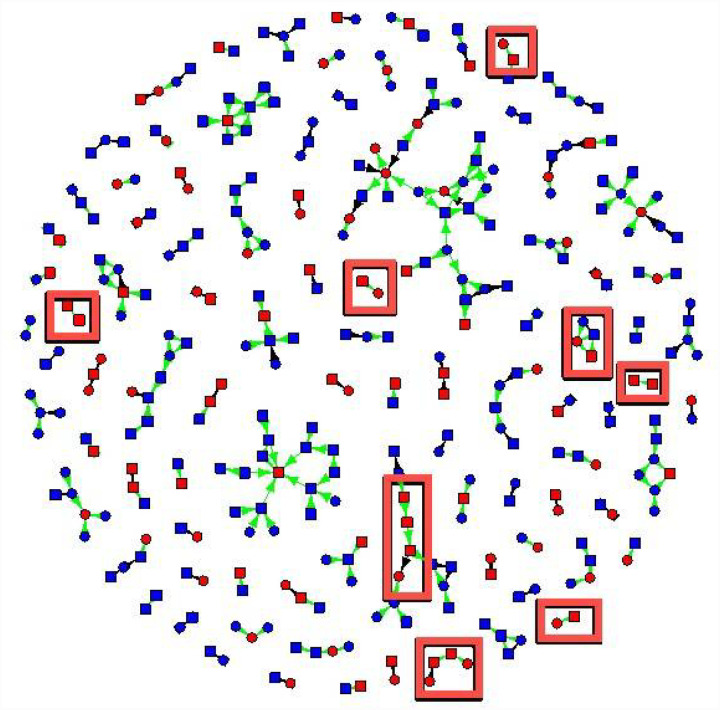
New cases of syphilis detected by eliciting in-person connections (red boxes). Syphilis status is coded by the color of vertices: red vertices tested positive for syphilis, and blue vertices tested negative for syphilis. Cases of syphilis that were detected due to asking for social ties are shown in red boxes. Squares are males, and circles are females. Green edges are social connections, and black edges are sexual connections. Arrows point from nominated individuals to index cases.

**Figure 2:**
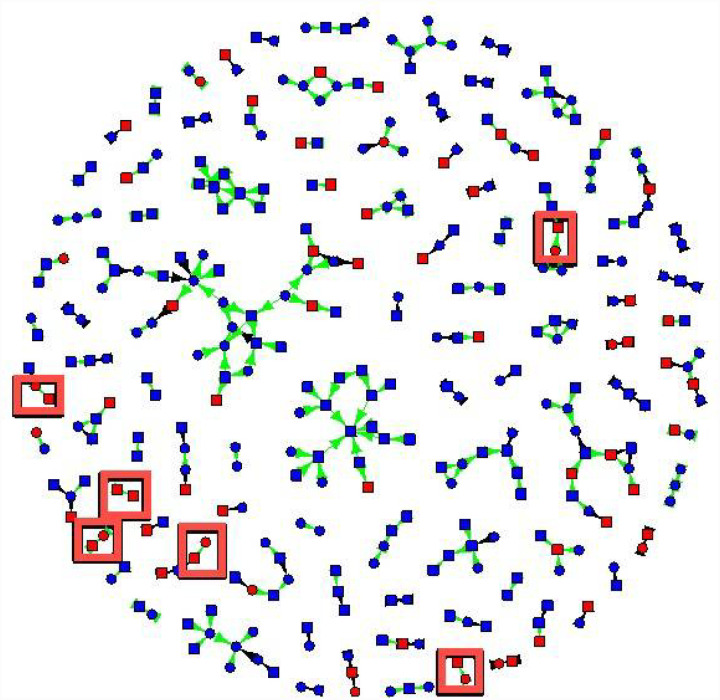
New cases of gonorrhea detected by eliciting in-person connections (red boxes). Gonorrhea status is coded by the color of vertices: red vertices tested positive for gonorrhea, and blue vertices tested negative for gonorrhea. Cases of gonorrhea that were detected due to asking for social ties are shown in red boxes. Squares are males, and circles are females. Green edges are social connections, and black edges are sexual connections.

The friendship network had 329 non-isolate members connected by 307 edges (Figures 1 and 2). The sexual network had 140 members connected by 100 edges. In several cases, two or more sexual dyads are connected by social connections. The largest network components have both sexual and social ties.

Figure 1 shows syphilis status, gender, and index versus contact status, and connections were coded as sexual versus social. For syphilis, 16 cases were connected by sexual contacts and 12 by social contacts (Figure 1). Participants with syphilis had 2.2 times the odds of having sexual contacts with syphilis as participants without syphilis (95% confidence interval (1.36, 3.66) and 1.3 times the odds of having social contacts with syphilis (95% CI 1.02, 1.68).

Figure 2 shows gonorrhea status, gender, and index versus contact status, and connections were coded as sexual versus social. For gonorrhea, four cases were connected by sexual contacts and six by social contacts (Figure 2). Participants with gonorrhea were not more likely to have sexual contacts with gonorrhea (AOR 0.98, 95% CI (0.62, 1.53), but they had 56% greater odds of having social contacts with gonorrhea (AOR 1.56, 95% CI (1.22, 2.00)).

Among index cases, 82.5% identified as straight, and 14% of males reported sex with another male (Table 2). However, only four males nominated other males as sexual contacts. Many more males nominated other males as social contacts. Many clusters of males include at least one male-male sexual partnership, which may imply that some of the other social contacts in these clusters of males are actually sexual.

We compared the demographic characteristics of index cases and their referred contacts. Compared with index cases, referred contacts were more likely to be straight-identified (92% vs. 83%), to have ever been married (31 % vs. 22%), to be Black (97% vs. 92%), and ages 45–60 years old (27% vs. 12%), and less likely to be male (50% vs. 72%), self-report MSM behavior (3% vs. 14%), to be employed full-time (25% vs. 39%), and ages 18–25 (20% vs. 31 %) (Table 2).

Participants with gonorrhea were younger on average (median 26 years old) than participants with syphilis (median 37 years old) and non-infected patients (median 38 years old); using a permutation test, we estimate p < 0.0001. Participants with syphilis or gonorrhea had slightly more sex partners in the past 90 days than non-infected participants (p=0.001 for syphilis; p=0.0025 for gonorrhea): the middle 90% of participants with syphilis had between 0 and 6 sexual partners in the past 90 days; the middle 90% of participants with gonorrhea had between 1 and 5 sexual partners; the middle 90% of non-infected participants had between 0 and 4 sexual partners. Participants with gonorrhea or syphilis did not differ in years of education from non-infected participants.

## 4. Discussion

This research shows that the friends of people diagnosed with gonorrhea and syphilis are also likely to have syphilis and gonorrhea, and that the syphilis strains between friends match according to the phylogenetic analysis. These findings suggest that friends are within the same sexual networks and/or have unreported sexual contact, not only homophily that friends engage in similar sexual risk behaviors. Further, eliciting friendships remains relevant despite the perception the mobile phone applications for seeking sexual partners have changed sexual habits because they facilitate sexual encounters outside usual social circles. However, research into how one mobile phone application is actually used found that 77% of users of one common application report seeking friendship, more than the percentages seeking dating (67%), one-on-one sex (62%), or group sex (17%) (16).

Including social connections increases number of connected individuals, size of networks, and same-sex connected individuals, and directly finds 12 syphilis and six gonorrhea cases. This improvement is comparable to the number of additional cases found per index case by using electronic communication methods such as e-mail, dating websites, and text messaging, compared with traditional contact-tracing methods such as using telephone and postal address (5). These findings cohere with findings that social network approaches improve HIV prevention interventions with hard-to-reach populations by identifying likely sexual network members (17, 18).

Males almost never referred to other males as sexual contacts, but males were likely to refer males as social contacts. These male-male connections may be friendships, or they may be sexual contacts between males who conceal their male-male sexual involvements. The high concordance in gonorrhea and syphilis status between social contacts may be because the index cases and their contacts interact in the same sexual networks but not directly connected by sexual involvement, or it could be because the index cases and contacts actually were sexually involved. The connection between cases was confirmed by the phylogenetic analysis of the syphilis strains, so these additional syphilis and gonorrhea cases are not completely explained by similar behaviors within friendship networks.

Eliciting social contacts in this study was done in person, rather than via electronic messaging, such as internet, text message, or mobile application. The 2008 recession led to budget cuts to public health departments that were not restored after the economic recovery, so disease intervention specialists have low resources, high caseloads, and high paperwork burden (2), so they may not have time to elicit social contacts as was done in this study. Future interventions could elicit social contacts via electronic messaging, but past intervention trials have found electronic messaging approaches to be greatly under- used by index cases (3,4), so eliciting social contacts via electronic messaging may also be as under-used as other methods of partner notification. Future investigations should also include a qualitative portion and input from a community advisory board so that social contacts are elicited to maximize positive impacts and minimize the potential for negative impacts.

The current intervention to elicit social contacts was developed in an urban environment in which people find both same and opposite sex sexual partners in their immediate vicinity (6). However, this intervention may not be as effective in rural areas where residents may find sexual partners at further distances; MSM and WSW may have particularly disparate partners either for reasons of stigma or partner availability. In both urban and rural areas, social contacts may be geographically close, but in rural areas, sexual partners may be more geographically dispersed than has been found in urban areas(6).

People may identify semi-anonymous sexual contacts at in-person “cruising” venues such as public restrooms, parks, bars or clubs, bathhouses, or via internet venues, such as Craigslist, internet chatrooms, or mobile phone applications such as Grindr (19–22). Technological change has created additional methods of meeting semi-anonymous partners, but it’s unclear whether technology has increased the number of semi-anonymous partners or substituted for in-person venues. Evidence from the earlier technological transition to the internet suggests that web-based personals substituted for in-person venues rather than increasing the total number of semi-anonymous contacts (20). Disease intervention specialists have difficulties identifying partners that index cases contacted only through partner-finding applications due to restrictions on using partner-finding applications for official government purposes(2). At the time of this study, many same-sex sexual contacts were facilitated by the internet, even without mobile telephone applications: a 2006 meta-analysis found 40% of MSM met partners on the internet and 30% had sex with someone they met on the internet (22), and 49% of rural males surveyed while attending urban Pride events in 2004–05 found partners via the internet (23). In 2002, 61 % of young MSM met their first male sexual partner on the internet, compared with 3% in 1993; as a corollary, young MSM became less likely to meet their first sexual partner at in-person venues, newspaper ads, or telephone chatlines between 1993 and 2002 (20). Since these data were collected for our study, computer-facilitated partnerships have shifted from both in-person and web-based venues to mobile phone applications (22).

Existing research has not established how frequently semi-anonymous sexual partners (e.g., from mobile applications or bathhouses) became in-person social contacts; if so, eliciting social contacts would elicit additional cases of STI even for index cases who do not admit same-sex activity. Although an unknown number of sexual contacts are semi-anonymous, so that sexual partners do not maintain a relationship after one or more sexual encounters, this study was able to identify 12 additional cases of syphilis and six additional cases of gonorrhea by eliciting in-person social contacts.

This approach to eliciting contacts yielded contacts who may be less likely to attend an STI clinic, such as people ages 45–60 years; among the contacts in this age group were four cases of syphilis, one case of gonorrhea, and one case of chlamydia.

## Strengths and limitations

This intervention was conducted among STI clinic patients in urban Baltimore, where half of sexual partners are located within 1 mile; in less urban and rural areas where sexual partners are more distant, social contacts might be less likely to be unidentified cases of gonorrhea and syphilis. Nonetheless, social contagion among friends has been found in less-urban areas, such as Framingham, MA (24), so social contacts of people with syphilis and gonorrhea may engage in more health risk behaviors, even if they are not concealed sexual contacts.

Seven index cases had neither gonorrhea nor syphilis were included in the study despite the inclusion criteria that index cases had to be diagnosed with gonorrhea or syphilis. These seven patients’ STI status may have been miscoded, or they may have been included by mistake. This measurement error or mistaken inclusion of STI negative participants biases results towards including STI negative contacts and does not represent a threat to the validity of this study.

The survey coded gender as only as male versus female, and gender was identified by the interviewer, rather than by the respondent. Although the interviewers were highly trained in interviewing related to HIV prevention interventions, this approach risks potentially misgendering participants. Sex assigned at birth was not assessed, so this study was not able to compare cis-gender and transgender individuals. Future studies should measure gender using self-report with inclusive survey items.

## Conclusions

Asking patients to name in-person social contacts in addition to sexual contacts may identify additional cases of gonorrhea and syphilis including from same-sex dyads, compared with asking sexual contacts only. Eliciting and contacting social contacts is more time-intensive than eliciting and contacting only sexual contacts. In the resurgence of syphilis through 2019, disease intervention specialists are prioritizing certain potentially pregnant women and HIV-positive patients due to low resources (25), so future interventions may need to develop methods to elicit in-person social contacts through electronic means that more index cases will use than past attempts (3,4).

The COVID-19 pandemic has spurred technology developers to develop technology protocols for privacy-preserving contact tracing that are anonymous, decentralized, encrypted, and automated, such as the Exposure Notification protocol developed by an Apple/Google collaboration (26). If these contact tracing protocols are successful in maintaining anonymity and maintain public trust, these protocols could be adapted for STI contact tracing. For example, existing mobile phone data are already used to identify users’ home and workplace locations (27). Combining these data with encrypted bluetooth and location data could be used to notify people with whom an infected person spends the most time outside their own home and workplace locations.

If a traditional approach to contact tracing is maintained, intervention trials can test whether index cases will be more likely to report both social and sexual contacts if they know that contacts will be offered self-administered mail-in tests or even self-testing resources for STIs that may be perceived as less intrusive, more accessible in rural areas, and more convenient than referral to clinics (28-32); internet-accessed STI testing may be particularly effective in reaching young adults who have never been tested for STI (33).

## Figures and Tables

**Table 1:** Twelve questions used to elicit friendship networks, followed by one question used to elicit sexual network. Respondents were asked to name first name and last initial or street name, and then asked later for other information for each including age, gender, relationship, trust, geographical proximity, frequency of contact, educational attainment, employment status, drug use, and connections between contacts. Every person named was followed by “Is there anyone else who you can think of?”

HEALTH ASSISTANCE
1. If you needed to go to the doctor, is there anyone you could call to take you there?
2. If you needed money to go to the doctor is there anyone you could ask to loan you or give you the money?
3. If you had to go to the hospital, who do you think would come to visit you while you were there?
4. If you were concerned that you might have a health problem is there someone you could talk to about it?
5. Is there anybody who you could ask for advice or help about health problems like: infections, gonorrhea, birth control, or AIDS?
SOCIAL PARTICIPATION
6. In the past six months: whom did you regularly eat meals with?
7. With whom did you share the same house, apartment or rooms?
8. Is there anybody that you could get together with to have fun, to relax, or to hang out with? These could be new names or ones you listed before.
9. (Asked only to alcohol drinkers) Who are the people that you drink with? These could be new names or ones you listed before.
MATERIAL AID
10. If you needed to borrow $25 or something valuable, is there anybody you know who would lend or give you $25, or more, or something that was valuable?
PERSONAL CONTACTS
11. If you wanted to talk to someone about things that are very personal and private or if a situation came up where you needed some advice, is there anybody you could you talk to?
12. Is there anybody who would give up some of their time and energy to help you - things like going with you someplace you needed to go, helping you do some work around the house, going to the store for you, and things like that? Remember that you might have listed these people before or they could be new names.
SEXUAL NETWORK
13. Of the people that you listed so far who did you have sex with in the last six months? Did you have sex with anyone else in the last six months? If yes, who?

**Table 2:** Demographic characteristics of index cases compared with contacts (both sexual and social).

	Index cases (%) (n=354)	Contacts (%) (n=172)	p-value
Male	72.0	50.0	<0.001
Age: under 18	3.7	2.3	0.4
Age 18–24 years	30.8	20.4	0.01
Age 25–34 years	22.0	19.2	0.5
Age 35–44 years	31.6	31.4	1.0
Age 45–60 years	11.9	26.7	<0.001
Straight-identified	82.5	92.4	0.002
MSM (among men)	14.2	2.6	0.006
WSM (among women)	99.0	95.7	0.2
Ever married	21.8	31.4	0.02
Employed full-time	38.9	25.4	0.002
Black or African-American	92.1	97.1	0.03
High school graduate	62.4	59.9	0.6
Imprisoned in past 10 years	35.3	36.6	0.8
Imprisoned in past 6 months	9.0	5.8	0.2
Injected drugs in past 10 years	12.7	18.6	0.07
Injected drugs in past 6 months	4.8	7.0	0.3

Percentages are percent of column. P-value is from a chi-square test and reported to 1 significant digit.

## Abbreviations

PN: partner notification
STI: sexually transmitted infection
OR: odds ratio
CI: confidence interval
MSM: men who have sex with men
WSW: women who have sex with women
COVID-19: Coronavirus Disease 2019
HIV: Human Immunodeficiency Virus
CDC: US Centers for Disease Control and Prevention
RPR: rapid protein reagin
FTA-ABS: Fluorescent Treponemal Antigen-Absorption test
RFLP: restriction fragment length polymorphism
MA: Massachusetts
